# Alteration of podocyte phenotype in the urine of women with preeclampsia

**DOI:** 10.1038/srep24258

**Published:** 2016-04-07

**Authors:** Tianyue Zhai, Itsuko Furuta, Rina Akaishi, Satoshi Ishikawa, Mamoru Morikawa, Takahiro Yamada, Takahiro Koyama, Hisanori Minakami

**Affiliations:** 1 Department of Obstetrics, Hokkaido University Graduate School of Medicine, Sapporo, Hokkaido, Japan

## Abstract

Podocyte injury has been suggested to induce phenotypic alteration of glomerular podocytes and accelerate the detachment of podocytes from the glomeruli resulting in podocyturia. However, it is not clear whether podocyte phenotypic alteration occurs in the urine of women with preeclampsia (PE). Seventy-seven and 116 pelleted urine samples from 38 and 18 women at various stages of normal and PE pregnancies, respectively underwent quantitative analysis of podocyte-specific or associated protein mRNA expression, including podocin, nephrin, and synaptopodin using RT-PCR. Significant proteinuria in pregnancy (SPIP) is defined as protein:creatinine ratio (P/Cr, mg/mg) ≥0.27 in the urine supernatant. All three urine-pellet mRNAs expression levels were significantly positively correlated with P/Cr levels, suggesting that podocyturia increased with proteinuria. The podocin:nephrin mRNA ratio (PNR) and synaptopodin:nephrin mRNA ratio (SNR) increased significantly with increasing P/Cr, while the podocin:synaptopodin mRNA ratio (PSR) did not change significantly according to P/Cr, resulting in significantly higher PNR and SNR, but not PSR levels, in urine from PE women with than without SPIP. The PNR, SNR, and PSR in urine from PE women before onset of SPIP were comparable to those from controls. Thus, nephrin mRNA expression was reduced in the podocytes recovered from PE women.

Podocytes are terminally differentiated cells that line the outer aspect of the glomerular basement membrane (GBM), and therefore form the final barrier to protein loss^1^. Podocytes detach from the GBM and are present in the urine of patients with various glomerular diseases, contributing to a decrease in number of podocytes at the GBM^2^. Podocyte loss at the GBM is associated with proteinuria, and podocyturia was suggested to occur earlier in the course of glomerular disease than proteinuria^3^. Podocyturia was confirmed in women with preeclampsia (PE) for the first time in 2007 by Garovic *et al*.^4^, and has been suggested to predate proteinuria and clinical features of PE^4^,^5^,^6^. Podocyturia was also reported in women with gestational hypertension, chronic hypertension, diabetes mellitus/gestational diabetes mellitus^7^, and uncomplicated pregnancies^8^, yielding new insight into the pathogenesis of proteinuria in pregnancy.

PE, a pregnancy-specific disorder occurring in approximately 3% of Japanese women^9^, is characterized by new onset of both hypertension and significant proteinuria in pregnancy (SPIP) on and after gestational week (GW) 20. Most PE women exhibit a gradual increase in proteinuria^10^. However, due to technical difficulties in quantification of podocyturia, it has not been determined whether podocyturia increases with increasing proteinuria in PE pregnancies.

Cell culture and immunohistochemistry for detection and quantification of podocyturia^4^,^6^ are laborious protocols requiring a great deal of time to obtain results and a significant level of expertise and training for interpretation of the results. Podocyte-specific proteins, such as nephrin and podocin that are known to contribute to the integrity of the filtration barrier, are markedly downregulated in the kidneys in nephrotic syndrome, and are helpful for podocyte identification^11^. Synaptopodin is a cytoskeletal protein present in the foot processes of podocytes, and its expression is also markedly diminished in the kidneys under proteinuric conditions^12^, although expression of synaptopodin mRNA is not specific for podocytes, occurring also in telencephalic dendrites^13^. These observations suggested that quantification of podocin (Pod-mRNA), nephrin (Nep-mRNA), and synaptopodin (Syn-mRNA) mRNA expression in pelleted urine samples may be useful for assessment of podocyturia.

In humans and animal models, podocyte loss in the kidney is associated with reduced nephrin mRNA expression in the urine podocytes, suggesting phenotypic alteration occurring in stressed or injured podocytes^14^,^15^,^16^. However, it is not yet known whether reduced nephrin mRNA expression occurs in the urine podocytes in PE pregnancy. The present study was performed to determine whether nephrin, podocin, and synaptopodin mRNA expression levels in the pelleted urine samples are correlated with the degree of proteinuria monitored by protein:creatinine ratio (P/Cr, mg/mg) in the urine supernatant and whether urinary expression levels of these genes are altered during the clinical course of PE pregnancy.

## Results

Of the 56 women included in the study, 38 remained normotensive throughout gestation and experienced uneventful normotensive pregnancies (Table 1). Gestational week (GW) at enrolment with the first urine sampling was significantly higher for PE women than for normotensive women. At the enrolment of 18 PE women, three had hypertension alone, six were diagnosed with PE, and the remaining nine women had neither hypertension nor SPIP. Thus, nine of the 18 women with PE were asymptomatic at the first urine sampling. The number of primiparous women, prevalence rate of preterm delivery at GW <37, and number of urine samples per woman were significantly greater, and GW at delivery and infant birthweight were significantly lower for PE women than for normotensive women (Table 1).

The levels of mRNAs encoding podocin, nephrin, synaptopodin, and glyceraldehyde 3-phosphate dehydrogenase (GAPDH) were examined in all 193 urine specimens from all 56 participants. In all of the samples, GAPDH expression was detectable, while at least one mRNA specific for podocytes was undetectable in 41 of the 193 urine specimens (21%) (Table 2). All three mRNAs specific for podocytes were detected in 98% (64/65) and 69% (88/128) or urine samples from women with and without SPIP (Table 2). However, as the threshold cycle (Ct) for GAPDH was significantly lower for urine samples from women with than without SPIP (21.2 [15.3–25.4] vs. 21.9 [17.8–25.2], respectively, *P* = 0.0167), the large number of cells, including podocytes, in pelleted urine samples from women with SPIP may have contributed to this difference. SPIP was absent in all 77 urine specimens from 38 normotensive women, and in 33 of 75 antepartum urine specimens (44%) and 18 of 41 postpartum urine specimens (44%) from 18 PE women (Table 2).

### Changes in Pod-mRNA, Nep-mRNA, and Syn-mRNA expression levels in normotensive (Fig. 1) and PE women (Fig. 2)

**Figure 1:**
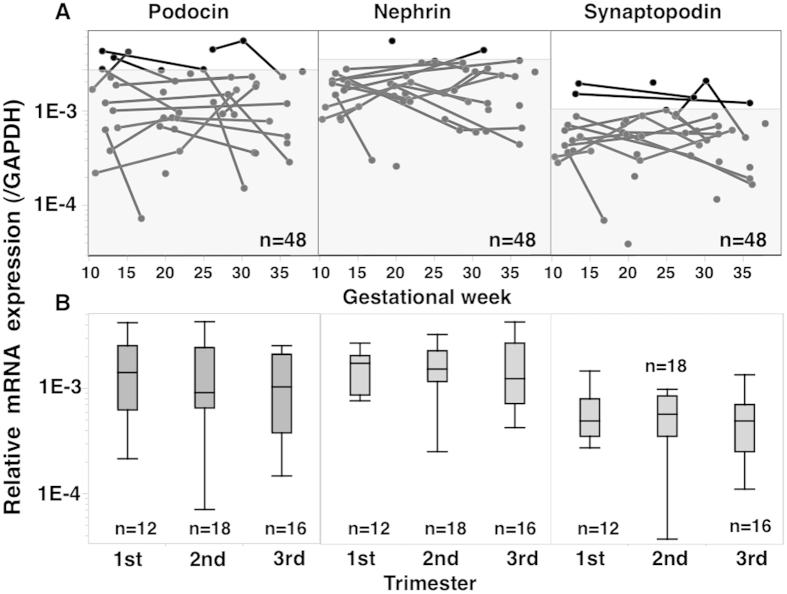
Changes in Pod-, Nep-, and Syn-mRNA expression levels in normtensive control pregnancies. In this analysis, only urine specimens showing detectable levels of all three mRNAs were used (*n* = 48). However, the 90^th^ percentile mRNA level was obtained using all 77 urine specimens from 38 normotensive control women on the assumption that in 29 specimens with at least one undetectable mRNA, all three mRNA expression levels were less than in 48 specimens with detectable levels of all three mRNAs. The shaded area in A indicates mRNA level <90^th^ percentile. (**B**) Changes in mRNA expression according to pregnancy trimester. Only one datum with the highest value was used for women with multiple urine samples available within the same pregnancy trimester. No significant changes in mRNA expression occurred during pregnancy in control women.

**Figure 2:**
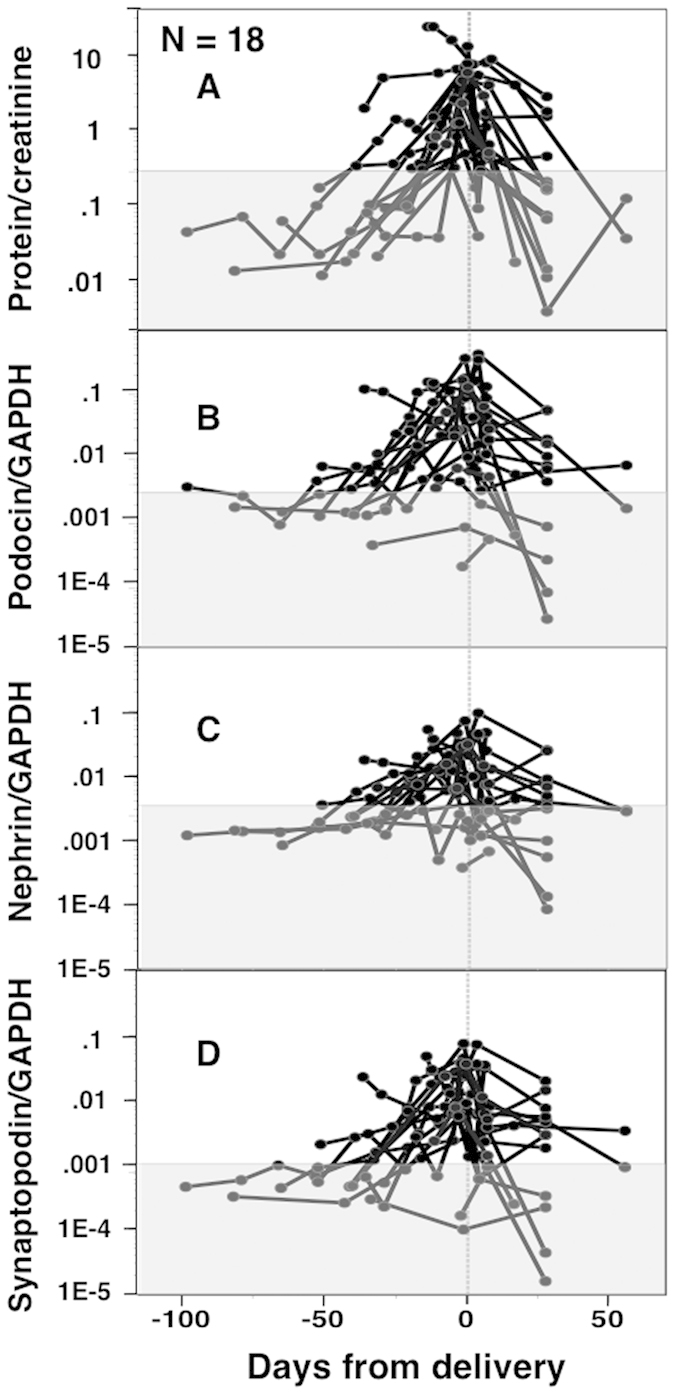
Perinatal changes in P/Cr level and mRNA expression in 18 PE women. In this analysis, only urine specimens showing detectable levels of all three mRNAs were used (*n* = 104). The shaded area indicates P/Cr <0.27 for (**A**) and <90^th^ percentile mRNA level determined in Fig. 1 for (**B**–**D**). Most PE women exhibited a gradual antenatal increase and postnatal decrease in P/Cr as well as the three mRNA expression levels.

There were no significant changes in expression of Pod-mRNA, Nep-mRNA, or Syn-mRNA in pelleted urine samples during pregnancies of normotensive control women (Fig. 1), while these transcripts showed increased expression with advancing gestation that decreased postpartum in PE women (Fig. 2B–D). The P/Cr level also appeared to increase with advancing gestation and decreased postpartum in PE women (Fig. 2A).

### Correlations of Pod-mRNA, Nep-mRNA, Syn-mRNA expression levels, PNR, SNR, and PSR with P/Cr levels in PE women (Figs 3 and 4)

**Figure 3:**
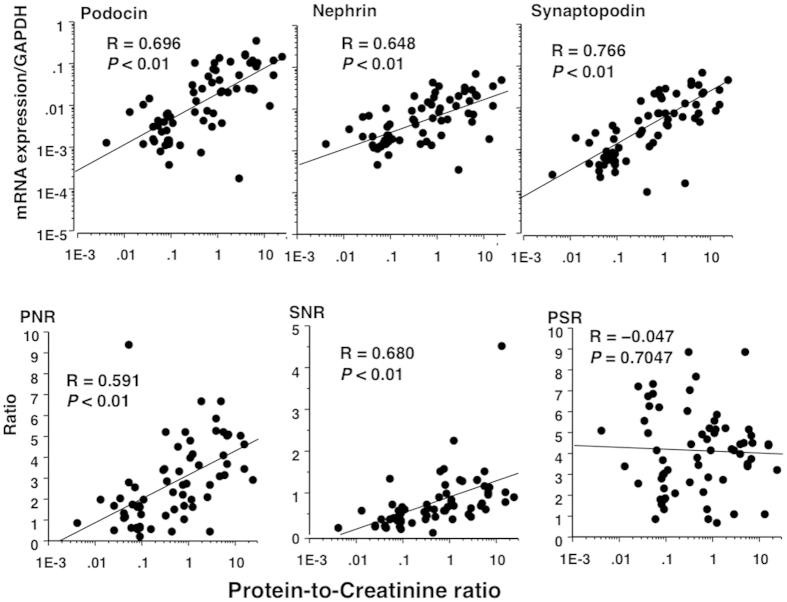
Association of P/Cr levels with three mRNA expression levels, PNR, SNR, and PSR in antenatal urine samples from PE women. In this analysis, only urine specimens showing detectable levels of all three mRNAs were used. In 65 antenatal urine samples from PE women, the relative expression levels of all three mRNAs (Pod-mRNA, Nep-mRNA, and Syn-mRNA) corrected by GAPDH were significantly positively correlated with P/Cr levels (upper panels). The PNR and SNR were also increased significantly with increasing P/Cr (lower panels). However, PSR did not change significantly according to P/Cr.

All three mRNA expression levels (corrected by GAPDH) were significantly positively correlated with P/Cr levels (Fig. 3, upper panels), suggesting that podocyturia (number of podocytes in the urine) increased with increasing proteinuria in PE women. Similarly, Pod-mRNA:Nep-mRNA ratio (PNR) and Syn-mRNA:Nep-mRNA ratio (SNR), but not Pod-mRNA: Syn-mRNA ratio (PSR), increased significantly with increasing P/Cr level (Fig. 3, lower panels). The PSR did not change significantly with P/Cr.

**Figure 4:**
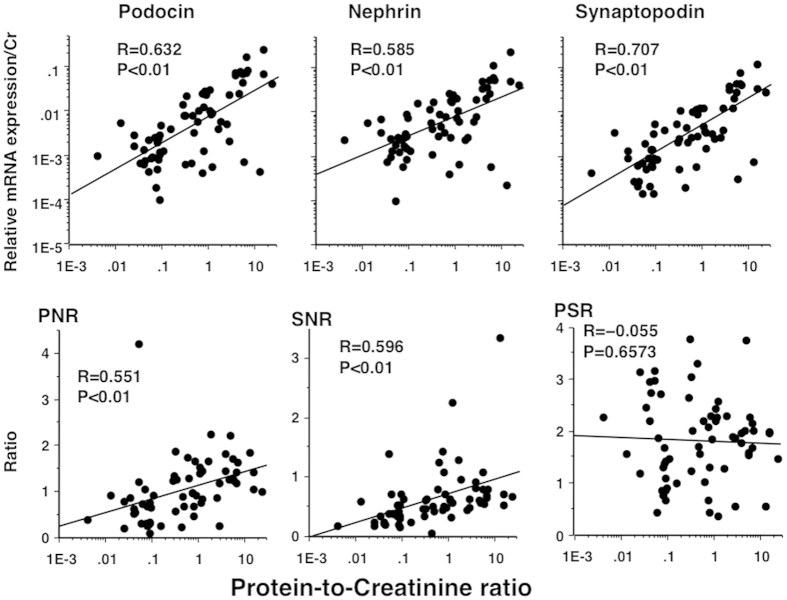
Association of P/Cr levels with three mRNA expression levels (corrected by urinary creatinine), PNR, SNR, and PSR in antenatal urine samples from PE women. Urine specimens were the same as those in Fig. 3. The relative expression levels of all three mRNAs were quantified using a standard curve method and corrected by creatinine weight (mg) contained in the whole sample. All correlations (indicated by R values) were somewhat weaker in this analysis than in the analysis for Fig. 3.

We had a concern in the inflammatory cells as another source of GAPDH in the pelleted urine samples in which the relative mRNA expression level to GAPDH was underestimated. However, the results shown in Fig. 3 were not altered greatly even after the relative expression of mRNAs was corrected by urinary creatinine (Fig. 4).

### Correlation of Pod-mRNA expression with Nep-mRNA and Syn-mRNA in tertile groups by P/Cr level in PE women (Fig. 5)

**Figure 5:**
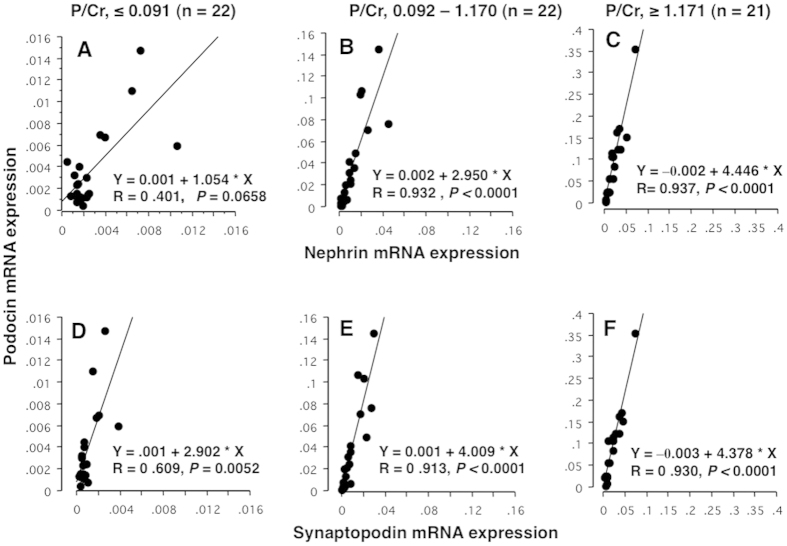
Association of Pod-mRNA expression with those of Nep- and Syn-mRNA in tertile groups by P/Cr level. Urine specimens were the same as those in Figs 3 and 4. Sixty-five antenatal urine samples from PE women were divided into tertile groups according to P/Cr (**A**,**D**, 1^st^ tertile group with P/Cr level ≤0.091; **B**,**E**, 2^nd^ tertile group with P/Cr level 0.092–1.170; and C and F, 3^rd^ tertile group with P/Cr level ≥1.171). (**A**–**C**) Correlations of Pod-mRNA against Nep-mRNA; (**D**–**F**, correlations of Pod-mRNA against Syn-mRNA. The changes in slope of linear regression equation according to P/Cr levels were of interest: 1.054, 2.950, and 4.446 for correlations between Pod-mRNA and Nep-mRNA of the 1^st^, 2^nd^, and 3^rd^ P/Cr tertile groups, respectively, and 2.902, 4.009, and 4.378 for correlations between Pod-mRNA and Syn-mRNA of the 1^st^, 2^nd^, and 3^rd^ P/Cr tertile groups, respectively. The degree of increase in correlation coefficient according to P/Cr was more marked for Pod-mRNA against Nep-mRNA than against Syn-mRNA. The correlation was very strong (R value >0.9) even between Pod-mRNA and Syn-mRNA for the 2^nd^ and 3^rd^ P/Cr tertile groups, suggesting that Syn-mRNA also derived from podocytes.

Antenatal urine samples from PE women were divided into tertile groups according to P/Cr (Fig. 5). Expression of Pod-mRNA was closely correlated with those of Nep-mRNA and Syn-mRNA at all P/Cr levels, especially in higher P/Cr ranges (correlation coefficient >0.9 for B, C, E, and F in Fig. 5) except for Podocin against Nephrin in the 1^st^ tertile group (Fig. 5). The correlation coefficient of linear regression equation appeared to increase at higher P/C ranges in both correlations between expression of Pod- and Nep-mRNA as well as Pod- and Syn-mRNA. However, the degree of increase in correlation coefficient was more marked for the correlation between Pod- and Nep-mRNA expression levels (Fig. 5, upper panels) than for Pod- and Syn-mRNA expression levels (Fig. 5, lower panels).

### Alterations of PNR, SNR, and PSR in relation to stages of PE pregnancies (Fig. 6)

**Figure 6:**
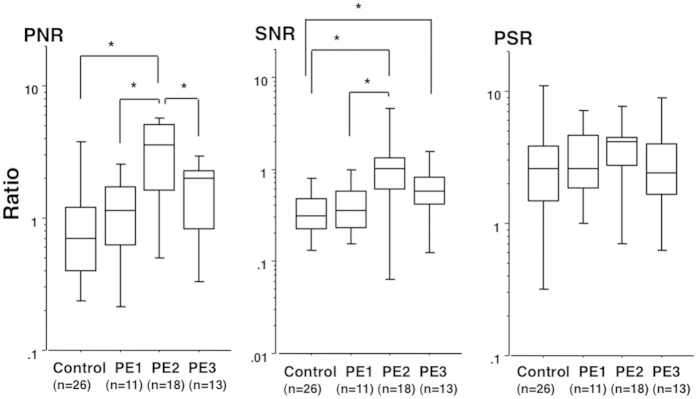
PNR, SNR, and PSR in control women and PE women in various stages of pregnancy. Urine samples from PE women were divided into three groups according to stage of PE pregnancy: PE1, antenatal but before SPIP onset; PE2, antenatal but after SPIP onset; and PE3, 1 month postpartum (collected on postpartum day 25–35). In this analysis, only urine specimens expressing detectable levels of all three mRNAs were used. Numbers in parentheses indicate the same number of urine specimens to number of individuals. Urine with the highest P/Cr level was used when multiple urine specimens meeting the designated condition were available for the same individual. The P/Cr levels were 0.06 (0.00–0.16), 0.07 (0.03–0.16), 3.82 (0.33–15.6), and 0.15 (0.01–2.14) for 26 control urine samples, 11 PE1 urine samples, 18 PE2 urine samples, and 13 PE3 urine samples, respectively. *P < 0.05 between two groups.

The levels of PNR and SNR, but not PSR, were significantly higher in antenatal urine samples from PE women with than without SPIP (Fig. 6). However, the PNR, SNR, and PSR levels in urine samples collected before the onset of SPIP were comparable to those in normotensive control women.

## Discussion

This study emphasized the following three points: (1) the expression levels of Pod-mRNA, Nep-mRNA, and Syn-mRNA in pelleted urine sample increased significantly with increasing proteinuria (monitored by P/Cr) and decreased postpartum in PE women; (2) PNR and SNR, but not PSR, increased with increasing proteinuria in PE women, resulting in significantly higher PNR and SNR levels, but not PSR levels, in the advanced stage of PE pregnancy compared to pre-clinical stage of PE pregnancy; and (3) the PNR, SNR, and PSR levels in urine samples from PE women before onset of SPIP were comparable to those in normotensive control women. These results suggested that in PE pregnancy, the number of podocytes detached from the GBM increased with increasing proteinuria and that the phenotype of urine podocytes of PE women was altered; podocytes with reduced Nep-mRNA expression were more likely to detach from the GBM and be excreted in the urine of PE women.

In this study, the expression levels of all three mRNAs in pelleted urine samples increased with increasing P/Cr (Figs 3 and 4) and were also correlated with each other (Fig. 5). Although the expression of synaptopodin is not specific for podocytes^13^, our results suggested that majority of urinary Syn-mRNA also derived from urinary podocytes. Generally, qPCR detects mRNA from viable or relatively intact cells, but it may also detect mRNA from apoptotic cells^17^. Therefore, the levels of mRNA expression in pelleted urine samples may have reflected the number of podocytes in urine, i.e., the degree of podocyturia, but not fragments of podocytes. These observations suggested that the number of podocytes detached from the GBM increased with increasing proteinuria. However, as the degree of increase in Nep-mRNA expression was less than that of Pod-mRNA, PNR increased markedly with increasing P/Cr (Figs 3 and 4). The degree of increase in Pod-mRNA was comparable to that in Syn-mRNA, resulting in no significant change in PSR according to P/Cr (Figs 3 and 4). These observations suggested that podocytes with reduced Nep-mRNA level were excreted in the urine of PE women, consistent with the results in animal models and human studies^14^,^15^; persistent proteinuria is associated with decreased Nep-mRNA expression^14^ and increased PNR in the urine of animal models and in urine from patients with SLE-associated glomerular disease^15^. In kidney biopsy specimens, expression of nephrin, but not podocin, is reduced in PE women^18^, and nephrin expression was shown to be reduced in the kidneys of women who died from PE^19^. These observations indicated that phenotypic alteration of glomerular podocytes occurred in PE pregnancy, and podocytes with reduced Nep-mRNA expression were likely to detach from the GBM.

Proteinuria monitored by P/Cr increases until delivery in PE pregnancy^10^, as was also confirmed in the present study, and decreases in proteinuria were seen postpartum in this study. Parallel to P/Cr, the levels of mRNAs changed in PE pregnancies. These observations suggested that the number of podocytes detached from the GBM continued to increase until delivery, and that delivery averted this process. As podocytes are terminally differentiated cells^1^ and their turnover rate is very low^20^,^21^, the detachment of podocytes from the GBM causes a long-lasting decrease in number of podocytes in the kidneys^2^. Indeed, the kidneys of PE women were suggested to have decreased numbers of podocytes^18^,^19^ and PE is a prominent risk factor for end-stage kidney disease (ESKD)^22^; among women with experience of three or more pregnancies, PE during one pregnancy is associated with a relative ESKD risk of 6.3 (95% CI, 4.1–9.9), and PE during two or three pregnancies is associated with a relative ESKD risk of 15.5 (95% CI, 7.8–30.8) compared to women with no PE pregnancies^22^. Aging is associated with a decrease in number of podocytes in the kidney^16^ and the risk of ESKD increases with age^23^, supporting the hypothesis derived from experiments in animal models that podocyte depletion in the kidney is associated with ESKD irrespective of the cause of kidney diseases^14^,^15^. PE pregnancy is a risk factor for ESKD possibly by the acceleration of renal glomerular podocyte depletion.

In this context, the glomerular podocyte depletion may explain why women with chronic hypertension are more likely to develop PE than normotensive women. SPIP develops and a diagnosis of PE is made in 10%–25% of women with chronic hypertension^24^,^25^,^26^, while approximately 3.0% of Japanese women develop both hypertension and SPIP and are diagnosed with PE^9^. Thus, women with chronic hypertension are several times more likely to exhibit SPIP than normotensive women. Chronic hypertension is a risk factor for ESKD^22^,^26^; among all of 570,433 Norwegian women with at least one experience of childbirth, ESKD developed in one in 1196 women approximately 17 years after the first pregnancy, while it developed in one of 90 women with chronic hypertension^22^. It was speculated that women with chronic hypertension may have already had a small number of glomerular podocytes even before the establishment of pregnancy, and therefore have been prone to SPIP. Pregnancy may have acted to further reduce the number of glomerular podocytes, subsequently leading to ESKD in some women with chronic hypertension.

In conclusion, Pod-mRNA, Nep-mRNA, and Syn-mRNA expression levels were determined using qPCR in pelleted urine samples from women with normal and PE pregnancies. All three mRNA expression levels increased with increasing amount of proteinuria in PE pregnancies, suggesting that the number of podocytes detached from the GBM continued to increase until delivery in PE pregnancies. The PNR and SNR, but not PSR, increased significantly with increasing proteinuria, suggesting that injured podocytes with reduced Nep-mRNA expression were excreted in the urine of PE women. This may have contributed to the podocyte loss in the kidneys of PE women and may explain why ESKD is more likely to occur in later life in women that have experienced PE pregnancy. Most current clinical guidelines for PE women do not include the amount of proteinuria as a criterion for consideration of early delivery. Further studies are required to determine the proteinuria level acceptable in continued pregnancy for reduction of future ESKD risk in PE women.

## Methods

This study was conducted in accordance with the principles expressed in the Declaration of Helsinki and with the approval of the Institutional Review Board of Hokkaido University Hospital, a tertiary teaching hospital managing mainly high-risk pregnant women.

### Participants

All 56 participants gave written informed consent prior to participation in this study. All women gave birth at Hokkaido University Hospital during the study period from May 2014 to May 2015, and provided spot urine specimens several times during pregnancy and postpartum. SPIP was defined as P/Cr ≥0.27 in the spot urine specimen. Hypertension was defined as the occurrence of systolic blood pressure ≥140 mmHg and/or diastolic blood pressure ≥90 mmHg on at least two occasions recorded more than 12 hours apart. A diagnosis of PE was made in women showing both hypertension and SPIP in the absence of known renal diseases. Women diagnosed with PE were admitted to the Department of Obstetrics of our hospital. Healthy pregnant women were monitored at the obstetric outpatient clinic of Hokkaido University Hospital.

### Urine Collection

Fifty-six women provided 193 spot urine specimens at various stages of pregnancy (Table 1). The number of urine samples per woman ranged from 1 to 12. All 193 urine samples were coded and processed anonymously within 2 hours of collection. Urine samples were transferred into tubes and centrifuged at 700 × *g* for 5 minutes. Urinary supernatant was stored at −20 °C until measurement of protein and creatinine levels using a Protein Assay Rapid Kit Wako and Laboassay Creatinine (Wako Pure Chemical Industries, Ltd., Osaka, Japan), respectively, to determine P/Cr. The pelleted urine samples were washed twice with phosphate buffered saline and centrifuged at 700 × *g* for 5 minutes. The pelleted urine samples were suspended in RNA*later* (Life Technologies, Carlsbad, CA, USA) and stored at −20 °C until isolation of RNA.

### Quantification of mRNA levels in the urine sediment

The RNA was isolated using the TRIzol method. The cell suspension in RNA*later* was centrifuged at 20000 × *g* for 3 minutes. Pellets were then dissolved with TRIzol (Life Technologies), and total RNA was extracted with RNeasy mini kits (Qiagen, Hilden, Germany) according to the manufacturer’s protocol. Following removal of contaminating genomic DNA with DNase, total RNA was purified using an RNeasy MinElute clean-up kit (Qiagen). Total RNA was quantified with a NanoDrop 1000 spectrophotometer (Thermo Fisher Scientific, Waltham, MA, USA). The ratio of absorbance at 260/280 nm was used to assess RNA purity. Reverse transcription of total RNA was performed using a PrimeScript RT Reagent kit (Takara Bio, Otsu, Japan) according to the manufacturer’s instructions.

Quantitative polymerase chain reaction (qPCR) was performed for assessment of urinary expression of Pod-mRNA, Nep-mRNA, and Syn-mRNA. The housekeeping gene, GAPDH, was used to normalize the mRNA expression level of each target gene. The following oligonucleotide primer sequences were used: podocin, sense 5′-AAGAGTAATTATATTCCGACTGGGACAT-3′ and antisense 5′-TGGTCACGATCTCATGAAAAGG-3′; nephrin, sense 5′-CAACTGGGAGAGACTGGGAGAA-3′ and antisense 5′-AATCTGACAACAAGACGGAGCA-3′; synaptopodin, sense 5′-AAGTCACATCCAGCTCCTTC-3′ and antisense 5′-CTTCTCCGTGAGGCTAGTG-3′; and GAPDH, sense 5′-GAAGGTGAAGGTCGGAGTC-3′ and antisense 5′-GAAGATGGTGATGGGATTTC-3′. Real-time PCR was performed using Power SYBR Green Master Mix (Invitrogen). The data were collected with an ABI Prism 7300 Sequence Detection System (Applied Biosystems, Foster City, CA, USA). The thermal cycling conditions were 95 °C for 10 minutes, followed by 40 cycles of 15 s at 95 °C and 1 minute at 60 °C. The relative quantification of target gene expression was performed using the 2^−ΔΔCt^ method. When a significant detectable increase in fluorescence did not occur in a specimen, we judged that the target gene was undetectable in that specimen.

### Statistical analyses

Data are presented as the median (range). Statistical analyses were performed using the JMP10^©^ statistical software package (SAS, Cary, NC, USA). The Wilcoxon/Kruskal–Wallis method was used for comparison of medians. The Mann–Whitney U test with Bonferroni’s correction was used to compare ratios of mRNA expression to P/Cr between the three groups. The Spearman’s rank-order correlation was used to test associations between two variables. In all analyses, *P* < 0.05 was taken to indicate statistical significance.

## Additional Information

**How to cite this article**: Zhai, T. *et al*. Alteration of podocyte phenotype in the urine of women with preeclampsia. *Sci. Rep.* **6**, 24258; doi: 10.1038/srep24258 (2016).

## Figures and Tables

**Table 1:** Demographic characteristics of 56 women.

	Normotensive	Preeclampsia
Number of women	38	18
Maternal age (year)	34 (24–42)	36 (19–43)
Primiparous	22 (58%)	15 (83%)*
At the enrollment		
GW	13.6 (11–39)	25.8 (15–36)*
Hypertension alone	None	3
Preeclampsia (PE)	None	6
Asymptomatic	38	9
Onset (GW)		
Hypertension	NA	30.5 (24–40)
SPIP	NA	30.5 (24–39)
GW at delivery	38 (36–41)	31.5 (26–40)*
<37	1 (2.6%)	12 (67%)*
Infant birthweight (kg)	3.00 (2.36–3.90)	1.47 (0.70–3.28)*
Total no. of urine samples	77	116
No. of samples/person	2 (1–3)	6.5 (2–12)*
Timing of urine sampling		
First trimester (5–13)	22 (28%)	0 (0%)
Second trimester (14–27)	26 (34%)	30 (26%)
Third trimester (28–40)	29 (38%)	45 (39%)
Postpartum	0 (0%)	41 (35%)

Data are presented as median (range). GW, gestational week; NA, not applicable; PE, preeclampsia; SPIP, significant proteinuria in pregnancy defined as protein-to-creatinine ratio (mg/mg) ≥0.27 in the spot urine specimen. *P < 0.05 vs. normotensive group. Forty-one postpartum urine specimens were collected on postpartum day 8 (1–60).

**Table 2:** Urine samples with at least one undetectable mRNA and P/Cr at that time.

	Were all three mRNA expression detected?	
	No	Yes
Overall, n = 193	41 (0.05 [0.01–0.38])	152 (0.09 [0.01–23.1]*)
Diagnosis of preeclampsia (PE)		
No, n = 77	29 (0.05 [0.01–0.15])	48 (0.03 [0.01–0.17])
Yes, n = 116	12 (0.05 [0.01–0.38])	104 (0.45 [0.01–23.1]*)
Presence of SPIP at urine sampling		
No, n = 128^†^	40 (0.05 [0.01–0. 25])	88 (0.04 [0.01–0.26])
Yes, n = 65	1 (0.38 [0.38–0.38])	64 (1.4 [0.28–23.1])

Number of urine samples with median P/Cr [range] in parenthesis is indicated in this table. SPIP, significant proteinuria defined as a P/Cr ≥0.27. *P < 0.05 vs. median P/Cr value in “No” group. ^†^SPIP was absent in all 77 urine specimens from 38 normotensive women, and in 33 of 75 antepartum urine specimens (44%) and 18 of 41 postpartum urine specimens (44%) from 18 PE women. All three mRNA expressions were detected in 65 (87%) and 39 (95%) of 75 and 41 antenatal and postnatal urine samples from PE women, respectively.
